# Whole genome sequencing reveals high clonal diversity of *Escherichia coli* isolated from patients in a tertiary care hospital in Moshi, Tanzania

**DOI:** 10.1186/s13756-018-0361-x

**Published:** 2018-06-08

**Authors:** Tolbert Sonda, Happiness Kumburu, Marco van Zwetselaar, Michael Alifrangis, Blandina T. Mmbaga, Frank M. Aarestrup, Gibson Kibiki, Ole Lund

**Affiliations:** 1 Kilimanjaro Clinical Research Institute, Kilimanjaro Christian Medical Centre, Moshi, Tanzania; 2 Kilimanjaro Christian Medical University College, Moshi, Tanzania; 3 Centre for Medical Parasitology, Department of Immunology and Microbiology, University of Copenhagen and Department of Infectious Diseases, Copenhagen University Hospital, Copenhagen, Denmark; 4 DTU-Food, Technical University of Denmark, Copenhagen, Denmark; 5 Centre for Biological Sequence Analysis, Technical University of Denmark, Copenhagen, Denmark; 6 East African Health Research Commission, Bujumbura, Burundi

**Keywords:** *E. coli*, Multi-locus sequence typing, Serotyping, And virulence, Whole genome sequencing, Tanzania

## Abstract

**Background:**

Limited information regarding the clonality of circulating *E. coli* strains in tertiary care hospitals in low and middle-income countries is available. The purpose of this study was to determine the serotypes, antimicrobial resistance and virulence genes. Further, we carried out a phylogenetic tree reconstruction to determine relatedness of *E. coli* isolated from patients in a tertiary care hospital in Tanzania.

**Methods:**

*E. coli* isolates from inpatients admitted at Kilimanjaro Christian Medical Centre between August 2013 and August 2015 were fully genome-sequenced at KCMC hospital. Sequence analysis was done for identification of resistance genes, Multi-Locus Sequence Typing, serotyping, and virulence genes. Phylogeny reconstruction using CSI Phylogeny was done to ascertain *E. coli* relatedness. Stata 13 (College Station, Texas 77,845 USA) was used to determine Cohen’s kappa coefficient of agreement between the phenotypically tested and whole genome sequence predicted antimicrobial resistance.

**Results:**

Out of 38 *E. coli* isolates, 21 different sequence types (ST) were observed. Eight (21.1%) isolates belonged to ST131; of which 7 (87.5.%) were serotype O25:H4. Ten (18.4%) isolates belonged to ST10 clonal complex; of these, four (40.0%) were ST617 with serotype O89:H10. Twenty-eight (73.7%) isolates carried genes encoding beta-lactam resistance enzymes*.* On average, agreement across all drugs tested was 83.9%. Trimethoprim/sulphamethoxazole (co-trimoxazole) showed moderate agreement: 45.8%, kappa =15% and *p* = 0.08. Amoxicillin-clavulanate showed strongest agreement: 87.5%, kappa = 74% and *p* = 0.0001. Twenty-two (57.9%) isolates carried virulence factors for host cells adherence and 25 (65.7%) for factors that promote *E. coli* immune evasion by increasing survival in serum. The phylogeny analysis showed that ST131 clustering close together whereas ST10 clonal complex had a very clear segregation of the ST617 and a mix of the rest STs.

**Conclusion:**

There is a high diversity of *E. coli* isolated from patients admitted to a tertiary care hospital in Tanzania. This underscores the necessity to routinely screen all bacterial isolates of clinical importance in tertiary health care facilities. WGS use for laboratory-based surveillance can be an effective early warning system for emerging pathogens and resistance mechanisms in LMICs.

**Electronic supplementary material:**

The online version of this article (10.1186/s13756-018-0361-x) contains supplementary material, which is available to authorized users.

## Background

*Escherichia coli* are Gram-negative bacterial commensals that naturally inhabit the human gastrointestinal tract (GIT). Through horizontal transfer and other mechanisms, commensal *E. coli* regularly acquire virulence, pathogenicity and multi-drug resistance properties from pathogenic *E. coli*. Consequently, *E. coli* is an important causative agent for a range of nosocomial and opportunistic infections including neonatal meningitis, diarrhoea, septicaemia, urinary tract and wound infections [1–5]. The global emergence and spread of multidrug resistant (MDR) *E. coli* in both community and as nosocomial infections, as well as in animals, warrants public health concerns [6–9]. Furthermore, virulent *E. coli* strains share resistance, virulence and pathogenic factors with avirulent or less virulent strains, enabling them to cause overlapping pathogenesis beyond their classical capacities [10]. Several *E. coli* outbreaks leading to serious health, social and economic impacts have been reported in high income countries (HICs) including the Netherlands [11], the UK [12], Norway, and Georgia [10].

Africa is highly burdened by diarrhoea and urinary tract infections (UTIs) that are *E. coli* related. For instance, in rural Kenya it was reported that 64.5% of UTI or bacteriuria was *E. coli* related [13]. In north-western Tanzania, 41.2 and 70% of UTI or bacteriuria was *E. coli* related among under-fives and febrile children, respectively [14, 15]. Similarly, in north-eastern part of Tanzania, *E. coli* accounted for 56.1% of all UTI cases among children [5] and *E. coli* accounted for 79 and 75% of UTI in non-malaria febrile children and adults, respectively [16]. In Dar es Salaam, two previous reports showed that, *E. coli* accounted for 51.1% of UTI in a survey done in 2010 [17] whereas as high as 64.0% of hospital- and community-acquired UTI was accounted for by *E. coli* in 2004 [18].

Finally, we recently showed that in Moshi, north-eastern Tanzania, *E. coli* was one of the most common bacterial pathogen isolates from a range of clinical manifestations [1]. Advanced molecular diagnostics such as whole genome sequencing (WGS) have revealed the emergence of a fatal diarrhoea-causing *E. coli* strain that combines virulence factors (VFs) from two *E. coli* strains [4]. The VFs are important properties that do enable an infectious agent to effectively and efficiently establish itself on or within its host by enhancing its potential to cause harm or disease. Some of the factors include those for host cell adherence, immune evasion, toxins and protease production for disrupting host cell pathways and scavenging minerals like iron in order to increase their survival. Certain *E. coli* strains have VFs that been linked to a serious outbreaks in China in 1999, where it caused 177 deaths [19], and in Germany in 2011, where it claimed 54 lives [20].

Molecular typing studies to evaluate *E. coli* related infections in low and middle-income countries (LMICs) are rare and little information is available regarding the specific subtypes causing infections in Tanzania or whether the high prevalence is due to sporadic infectious or clonal transmission between patients. The purpose of this study was to determine the serotypes, antimicrobial resistance and virulence genes. Further, we carried out a phylogenetic tree reconstruction to determine relatedness of *E. coli* isolated from patients in a tertiary care hospital in Tanzania using the WGS-based diagnostic platform installed at KCMC hospital in Moshi, Tanzania.

## Methods

### Study design, participants and specimen collection

A hospital based prospective cross-sectional study was conducted at KCMC between August 2013 and August 2015. Part of the study’s methods has been described in details by Kumburu et al. [1]. KCMC is located in Moshi municipality, north-eastern Tanzania and serves as a zonal referral hospital for a catchment area of around 15 million people. The hospital has a bed capacity of 650 with approximately 500 outpatients seeking medical services daily. This study was granted ethical approval by the KCMC Research Ethics Committee and the National Institute for Medical Research. A written informed consent was obtained from each participant or from parents or guardians of children before enrolment into the study. The study involved patients admitted in medical, surgical and paediatrics wards who were suspected to have bacterial infection. Specimens collected for bacterial culture were sputum, wound or pus swab, stool and blood. Bacteria culture, isolation, and identification were performed according to in-house standard operating procedures and the Clinical and Laboratory Standards Institute (CLSI) guidelines as described by Kumburu et al. [1]. Over a 2-year period, 590 samples were collected without apriori knowledge of the infecting agent. A total of 377 bacterial strains were isolated, and whole genome sequenced. A number of isolates from this collection were randomly selected for antimicrobial susceptibility testing. A total of 38 *E. coli* collected sequentially were included in this study; among which 24 *E. coli* isolates had antimicrobial susceptibility results.

### Genomic DNA isolation, whole genome sequencing, and analysis

For all *E. coli* isolates genomic DNA (gDNA) was purified and its concentration determined using the Easy-DNA extraction kit (Invitrogen®) and the Qubit dsDNA Assay Kit (Invitrogen®), respectively. The gDNA library preparation was performed following Nextera® XT DNA Sample Preparation Guide [21]. In brief, each gDNA was tagmented (tagged and fragmented) by the Nextera® XT transposome. The transposome simultaneously fragments the input DNA and adds adapter sequences to the fragment ends. Then followed a limited-cycle PCR amplification whereby indexes required for cluster formation were added to each DNA piece. Then each gDNA library was normalized to ensure equal representation during sequencing. Equal volumes of the normalized library were combined, diluted in hybridization buffer, and heat denatured prior to sequencing on the Illumina MiSeq platform (Illumina Inc.). The sequencer output was analysed using the standard WGS pipeline at KCRI, which is based on local implementations of the bioinformatics services available at https://cge.cbs.dtu.dk/services/. Quality control of the reads was performed using FastQC 0.11.4 [22]. De novo assembly was performed with SPAdes 3.11.1 [23], and quality assessed using QUAST 4.5 [24]. For this article’s purpose the analyses included: resistance genes identification using ResFinder 2.1 [25], Multi-Locus Sequence Typing (MLST) determination using MLST 1.8 [26], serotyping using SeroTypeFinder 1.1 [27], and virulence genes determination using VirulenceFinder 1.4 [28]. Phylogeny reconstruction was done using CSI Phylogeny [29] (with reference strain EC958, NZ_HG941718.1). The 38 assembled *E. coli* genomes of the present study have been submitted to the European Nucleotide Archive with project accession number PRJEB23541. The phylogenetic analyses included 6 more *E. coli* genomes previously isolated from animals in Mwanza, north-western Tanzania, by Seni et al. [30]. The raw sequence data of the *E. coli* of animal origin were downloaded from the European Nucleotide Archive (ENA) under the project number PRJEB12335. Stata 13 (College Station, Texas 77,845 USA) was used to determine Cohen’s kappa coefficient of agreement between the phenotypically tested and whole genome sequence predicted antimicrobial resistance.

## Results

### Specimens and isolates

A total of 38 non-duplicate *E. coli* were isolated, of which 18 (47.4%) were from wound (or pus) swabs, 13 (34.2%) from diarrhoeal stool, 4 (10.5%) from sputum and 3 (7.9%) from blood. Twenty-three (60.5%) were isolated from patients in medical wards, 9 (23.7%) from patients in surgical wards, and 6 (15.8%) from patients in intensive care unit (ICU). Out of these six *E. coli* isolates, four were from surgical ICU and 2 from medical ICU. Thirteen (34.2%) *E. coli* were isolated in 2013, 16 (42.1%) in 2014 and nine (23.7%) in 2015 (Table 1).

**Table 1:** Origin and drug susceptibility results of 38 clinical *E. coli* isolates

Origin of isolate				Drug susceptibility results^d^									
Isolate	ward^b^	specimen^c^	year^a^	AMC	AM	CZ	CAZ	CRO	C	CIP	GM	NA	SXT
4	MW	Stool	2013	S	R	S	S	S	S	S	S	S	R
5	MW	Stool	2013	S	S	S	S	S	S	S	S	S	S
6	MW	Stool	2013	S	S	S	S	S	S	S	S	S	S
10	SW	Swab	2013	R	R	S	S	S	S	R	S	R	S
21	MW	Stool	2013	R	S	S	S	S	S	S	S	S	R
22	MW	Stool	2013	N	N	N	N	N	N	N	N	N	N
30	SW	Swab	2013	S	S	S	S	S	S	S	S	S	S
44	SICU	Swab	2013	S	R	R	R	R	S	R	R	R	R
70	MW	Sputum	2013	R	R	R	R	R	S	R	S	R	R
73	MW	Stool	2013	R	R	R	R	R	S	R	S	R	R
82	MW	Stool	2013	R	R	R	S	R	S	R	S	R	R
97	MW	Stool	2014	N	N	N	N	N	N	N	N	N	N
115	SW	Swab	2014	S	R	R	R	R	S	S	S	S	R
199	MW	Blood	2014	R	R	R	R	R	R	R	R	R	R
203	MW	Stool	2014	R	R	S	S	S	S	R	S	R	R
210	MW	Blood	2014	R	R	R	R	R	S	R	R	R	R
244	MW	Sputum	2014	R	R	R	R	R	S	R	R	R	R
245	MW	Swab	2014	R	R	R	R	R	S	R	R	R	R
247	MW	Blood	2014	R	R	R	S	R	S	R	S	R	R
298	MW	Stool	2014	N	N	N	N	N	N	N	N	N	N
365	SICU	Swab	2014	R	R	R	R	R	S	R	S	R	R
393	MICU	Stool	2015	R	R	R	R	R	S	R	R	R	R
521	MW	Sputum	2014	S	S	S	S	S	S	S	S	R	R
538	SW	Swab	2015	N	N	N	N	N	N	N	N	N	N
554	MW	Stool	2015	N	N	N	N	N	N	N	N	N	N
587	SW	Swab	2015	N	N	N	N	N	N	N	N	N	N
603	MICU	Swab	2015	N	N	N	N	N	N	N	N	N	N
118A	SW	Swab	2014	S	R	S	S	S	S	S	S	S	S
119EC	MW	Stool	2014	S	S	R	R	R	R	S	S	S	S
163A	SW	Swab	2014	S	S	S	S	S	S	S	S	S	S
237C	SW	Swab	2014	N	N	N	N	N	N	N	N	N	N
340B	MW	Sputum	2014	S	S	S	S	S	S	S	S	S	R
431D	SICU	Swab	2015	N	N	N	N	N	N	N	N	N	N
567B	SW	Swab	2015	N	N	N	N	N	N	N	N	N	N
598A	MW	Swab	2015	N	N	N	N	N	N	N	N	N	N
598B	MW	Swab	2015	N	N	N	N	N	N	N	N	N	N
71E	MW	Swab	2013	R	R	R	R	R	S	R	R	R	R
77E	SICU	Swab	2013	R	R	R	R	R	S	R	S	R	S

^a^year of collection

^b^ *MW* Medical ward, *SW* surgical ward, *MICU* medical ICU, *SICU* surgical ICU

^c^Wound or pus swab, diarrhoea or stool

^d^ *S* Susceptible, *R* Resistant, *N* Not tested, *AMC* Amoxicillin-Clavulanate**,** *AM* ampicillin**,** *CZ* cefazoline**,** *CAZ* ceftazidime, *CRO* ceftriaxone**,** *C* chloramphenicol**,** *CIP* ciprofloxacin, *GM* gentamycin, *NA* Nalidixic acid, *SXT* trimethoprim sulphamethoxazole

### Multi-locus sequence typing and serotyping

Out of 38 *E. coli* isolates, 21 different STs were observed. Eight (21.1%) *E. coli* belonged to ST131; 3 isolated in 2013, 2 in 2014 and 3 in 2015 (Table 1). Two of the *E. coli* ST131 were isolated from patients in surgical wards, and six from patients in medical wards, including one from medical ICU. Of these eight, seven (87.5%) *E. coli* had serotype O25:H4 and one had serotype O15:H1. Out of 38 isolates, 10 (18.4%) belonged to ST10 clonal complex; of these, four (40.0%) were ST617 with serotype O89:H10, of which 3 were isolated in 2014 and 1 in 2015 and three (30.0%) were ST10, of which two had serotype O89:H10 and one had serotype O89:H9 (Table 2). These ST10 were all isolated in 2014 from medical wards. We noted that, out of the three ST410, one had serotype O8:H21, and the others had unknown O-group but belonged to H9.

**Table 2:** Sequence types, Serotypes and Virulence factors of 38 clinical *E. coli* isolates

Isolate	ST^e^	Serotype	Virulence Factors				
			Adherence	Toxin	Protease	Evasion^g^	Type III^h^
70	ST-131	O25:H4	*Iha,nfaE*		*sat*	*iss*	
73	ST-131	O25:H4	*Iha,nfaE*		*sat*	*iss*	
199	ST-131	O25:H4	*Iha*	*astA,senB*	*sat*	*iss*	
210	ST-131	O25:H4	*Iha,nfaE*	*senB*	*sat*	*iss*	
587	ST-131	O25:H4	*Iha*		*sat*	*iss*	
603	ST-131	O15:H1	*iha,nfaE lpfA,eilA*	*senB*	*sat*	*iss*	*air*
567B	ST-131	O25:H4	*Iha*		*sat*	*iss*	
71E	ST-131	O25:H4	*iha,nfaE*	*senB*	*sat*	*iss*	
244	ST-617^f^	O89:H10				*iss*	
245	ST-617 ^f^	O89:H10				*iss*	
538	ST-617 ^f^	O89:H10		*astA,senB*		*iss*	*capU*
237C	ST-617 ^f^	O89:H10				*iss*	
393	ST-405	O102:H6	*eilA*				*air*
10	ST-410	O??:H9	*lpfA*				
22	ST-410	O??:H9	*lpfA*				
598B	ST-410	O8:H21	*lpfA*			*iss*	
97	ST-10 ^f^	O89:H10					
203	ST-10 ^f^	O89:H10					
340B	ST-10 ^f^	O89:H9				*iss*	
44	ST-167 ^f^	O89:H21					
247	ST-167 ^f^	O89:H9		*senB*		*iss*	*capU*
5^a^	ST-226	O40:H19	*bfpA,eae*		*espA,espF,espJ*		*nleB,nleC*
6^a^	ST-226	O40:H19	*bfpA,eae*		*espA,espF,espJ*		*nleB,nleC*
118A^d^	ST-73	O6:H1	*Iha*	*pic*	*sat,vat*	*iss*	*mchB,mchC,mchF,mcmA*
21	ST-942	O39:H28	*lpfA*			*iss*	
554^c^	ST-95	O2(50):H4				*iss*	*mchF*
431D	ST-44 ^f^	O89:H4					
119EC	ST-4959	O154:H4					
521^b^	ST-504	O166:H7	*Iha*		*vat*	*iss*	*mchB,mchC,mchF,mcmA, sigA, capU*
4	ST-2332	O128:H45	*cfaC, lngA*		*eatA*		
30	ST-355	O150:H5		*pic*	*vat*	*iss*	
115	ST-361	O9:H30					
163A	ST-372	O83:H31			*vat*	*iss*	
298	ST-38	O86:H18	*Iha,nfaE eilA*	*senB*	*sat*	*iss*	*air*
365	ST-224	O8:H23	*lpfA*			*iss*	
82	ST-156	O61:H34	*lpfA*			*iss*	
598A	ST-1193	O75:H5	*Iha*	*senB*	*sat,vat*		
77E	ST-1284	O89:H21				*iss*	

^a^Has virulence factor *tir*

^b^Has virulence factor *iroN*

^c^Has virulence factors *ireA,iroN*

^d^Has virulence factor *ireA*

^e^Sequence Type (ST)

^f^ST-10 clonal complex

^g^Immune evasion

^h^Type III translocated protein

### Virulence factors

Table 2 as well show the distribution of virulence factors (VFs) among the sequenced *E. coli* isolates. A total of 22 (57.9%) *E. coli* isolates carried VFs for host cells adherence. These included 12 (31.6%) for *iha*, 7 (18.4%) for *lpfA* and 6 (15.8%) for each of *aafC* and *nfaE*. The prevalence of *eilA* was 3 (7.9%) and 2 (5.3%) for each of *eae* and *bfpA*. A total of 9 (23.7%) *E. coli* isolates had VFs that promote toxin production. The distribution of VFs that promote toxin was 7 (18.4%) for *senB* and 2 (5.3%) for each of *astA* and *pic*. The VFs that promote *E. coli* protease production were detected in 18 (47.4%) *E. coli* isolates. The prevalent protease VFs were *sat* and *vat* with 13 (34.2%) and 5 (13.2%), respectively. A total of 25 (65.7%) *E. coli* isolates had *iss*, a factor that promotes *E. coli* immune evasion by increasing serum survival.

### Acquired antimicrobial resistance

A total of 28 (73.7%) *E. coli* carried genes encoding beta-lactam resistance enzymes (Table 3)*.* The bla_CTX*-M-15*_ was harboured by 17 (44.7%) isolates*, bla*_*OXA-1*_ harboured by 19 (50%) and *bla*_*TEM*-1B_ by 12 (31.6%). The overall proportion of *dfrA* genes encoding trimethoprim resistance enzymes was 28 (73.7%). The *dfrA17* was the most abundant gene with a proportion of 16 (42.1%) followed by 6 (15%) for *dfrA14* genes, and 2 (5.3%) for each of *dfrA5, dfrA7* and *dfrA12*.

**Table 3:** Acquired antimicrobial resistance genes of 38 clinical *E. coli* isolates

Isolate	AMG^a^	BL^b^	FQA^c^	Macrolide	Phenicol	Quinolone	Sulphonamide	Tetracycline	Trimethoprim
4	*strA strB*	bla_TEM-1B_					*sul2*		*dfrA14*
5									
6									
10		bla_OXA-1_	*aac(6’)Ib-cr*		*catB3*			*tet(A)*	
21	*strA strB*						*sul2*	*tet(A)*	*dfrA14*
22		bla_OXA-1_	*aac(6’)Ib-cr*		*catB3*			*tet(A)*	
30									
44	*aac(3)-IId aadA2*	bla_CTX-M-15_ bla_TEM-1B_		*mph(A)*	*catA1*	*QnrS1 qepA QnrD*	*sul1*	*tet(A)*	*dfrA12*
70	*aadA5*	bla_CTX-M-15_ bla_OXA-1_	*aac(6’)Ib-cr*	*mph(A)*	*catB3*		*sul1*	*tet(A)*	*dfrA17*
73	*aadA5*	bla_CTX-M-15_ bla_OXA-1_	*aac(6’)Ib-cr*	*mph(A)*	*catB3*		*sul1*	*tet(A)*	*dfrA17*
82	*strA strB aadA2*	bla_TEM-1B_		*mph(A)*			*sul2 sul1*	*tet(B) tet(A)*	*dfrA12*
97		bla_OXA-1_	*aac(6’)Ib-cr*		*catB3*			*tet(B)*	
115	*aadA1 aac(3)-IIa strB strA*	bla_CTX-M-15_ bla_TEM-1B_ bla_OXA-1_	*aac(6’)Ib-cr*		*catB3 catA1*	*QnrB1*	*sul2*		*dfrA14*
163A									
199	*aadA1 strB strA aadA5 aadB*	bla_CTX-M-15_ bla_TEM-1B_ bla_OXA-1_	*aac(6’)Ib-cr*	*mph(A)*	*catB3 cmlA1*		*sul1 sul2*	*tet(A)*	*dfrA17*
203	*strB strA*	bla_TEM-1C_ bla_OXA-1_	*aac(6’)Ib-cr*		*catB3*		*sul2*	*tet(A)*	*dfrA14*
210	*aadA5 aac(3)-IIa strB strA*	bla_CTX-M-15_ bla_OXA-1_	*aac(6’)Ib-cr*	*mph(A)*	*catB3*		*sul1 sul2*	*tet(A)*	*dfrA17*
237C	*aac(3)-Iia aadA5 strA strB*	bla_CTX-M-15_ bla_OXA-1_	*aac(6’)Ib-cr*	*mph(A)*	*catB3*		*sul1 sul2*	*tet(B)*	*dfrA17*
244	*aac(3)-IIa aadA5 strA strB*	bla_CTX-M-15_ bla_OXA-1_	*aac(6’)Ib-cr*	*mph(A)*	*catB3*		*sul2 sul1*	*tet(B)*	*dfrA17*
245	*strA aac(3)-IIa strB aadA5*	bla_CTX-M-15_ bla_OXA-1_	*aac(6’)Ib-cr*	*mph(A)*	*catB3*		*sul1 sul2*	*tet(B)*	*dfrA17*
247	*strA strB aadA5*	bla_OXA-1_ bla_CTX-M-15_	*aac(6’)Ib-cr*	*mph(A)*	*catB3*		*sul2 sul1*	*tet(A)*	*dfrA17*
298	*strB strA*	bla_TEM-1B_			*catA1*		*sul2*	*tet(D)*	*dfrA7*
365	*aadA5*	bla_CTX-M-15_ bla_OXA-1_	*aac(6’)Ib-cr*	*mph(A)*	*catB3*		*sul1*	*tet(A)*	*dfrA17*
393	*aac(3)-Iia aadA5*	bla_CTX-M-15_ bla_OXA-1_	aac(6’)Ib-cr	*mph(A)*	*catB3*		*sul1*	*tet(B)*	*dfrA17*
431D	*erm(B) strA strB*						*sul2*		
521							*sul2 sul1*		*dfrA5*
538	*aac(3)-Iia aadA5 strA strB*	bla_CTX-M-15_ bla_OXA-1_	*aac(6’)Ib-cr*	*mph(A)*	*catB3*		*sul1 sul2*	*tet(A) tet(B)*	*dfrA17*
554	*strA strB*	bla_TEM-1B_					*sul2*		*dfrA5*
567B	*aadA5*			*mph(A)*			*sul1*		*dfrA17*
587	*aadA5*	bla_CTX-M-15_		*mph(A)*			*sul1*		*dfrA17*
603	*strB aadA5 aac(3)-IId strA*	bla_TEM-1B_		*mph(A)*			*sul2 sul1*		*dfrA17*
118A	*strB strA*	bla_TEM-1B_					*sul1 sul2*		*dfrA7*
119EC									
340B	*strB strA*						*sul2*	*tet(A)*	*dfrA14*
598A	*strB strA*	bla_TEM-1B_		*mph(A)*			*sul2*	*tet(B)*	*dfrA17*
598B	*aac(3)-IIa strB strA*	bla_TEM-1B_ bla_CTX-M-15_ bla_OXA-1_	*aac(6’)Ib-cr*	*mph(A)*	*catB3*		*sul2*	*tet(A)*	*dfrA14*
71E	*aadA5 aac(3)-IIa strB strA*	bla_CTX-M-15_ bla_OXA-1_	*aac(6’)Ib-cr*	*mph(A)*	*catB3*		*sul1 sul2*	*tet(A)*	*dfrA17*
77E	*strA strB*	bla_CTX-M-15_ bla_OXA-1_	*aac(6’)Ib-cr*		*catB3*		*sul2*	*tet(B)*	

^a^ Aminoglycoside

^b^ Beta-Lactam

^c^ Fluoroquinolones and aminoglycoside

A total of 19 (50%) *E. coli* carried *mph(A)*, a gene encoding macrolide resistance enzymes including towards erythromycin and azithromycin*.* The prevalence of genes encoding sulphonamides resistance enzyme for *sul1* and *sul2* were 19 (47.5%) and 24 (60.5%), respectively*.* The proportion of genes encoding tetracycline resistance enzymes for *tet(A)* and *tet(B)* were 16 (42.1%) and 9 (23.7%) respectively. The proportions of genes encoding chloramphenicol resistance enzymes were 19 (50%) for *catB3* and 3 (7.9%) *for catA1.* A total of 22 (57.9%) isolates carried both *strA* and *strB* genes against aminoglycoside. The gene *aadA5* was detected in 15 (39.5%), for each *aadA2* and *aadA1* in 2 (5.3%) and *aadB* in 1 (2.6%) *E. coli* isolates. Others included *aac(3)-IIa* in 9 (23.7%) and *aac(3)-IId* in 2 (5.3%) of the *E. coli* isolates.

### Phenotype and sequence based antimicrobial resistance comparison

Agreement between phenotype and whole genome sequence based antimicrobial resistance was done for 24 out of 38 *E. coli* isolates (Table 4). On average, agreement across all drugs tested was 83.9%. Overall, the phenotypically determined resistance was higher than sequence-based resistance. However, all but trimethoprim sulpha or co-trimoxazole showed strong agreement (81–100%) between phenotype and sequence-based resistance results. Trimethoprim/sulphamethoxazole (co-trimoxazole) showed moderate agreement: 45.8%, kappa =15% and *p* = 0.08. Sequence-based analysis predicted resistance in 4 (16.7%) isolates, whereas phenotypic testing revealed 17 (70.8%) isolates to be resistant. Amoxicillin-clavulanate showed strongest agreement: 87.5%, kappa = 74% and *p* = 0.0001. Sequence-based analysis predicted resistance to amoxicillin-clavulanate in 14 (58.3%) isolates, whereas 15 (62.5%) isolates were found to be resistant phenotypically.

**Table 4:** Agreement between phenotypically tested and whole genome sequence predicted antimicrobial resistance

Antibiotic name	DST^a^	WGS^b^	Agreement	Kappa	*P* value
Amoxicillin-Clavulanate	15 (62.5%)	14 (58.3%)	0.875	0.74	0.0001
Ampicillin	16 (66.7%)	14 (58.3%)	0.9167	0.82	0.00
Ceftazidime	13 (54.2%)	13 (54.2%)	0.9167	0.83	0.00
Ceftriaxone	15 (62.5)	13 (54.2%)	0.9167	0.84	0.00
Chloramphenicol	2 (8.3%)	2 (8.3%)	0.8333	−0.09	0.672
Ciprofloxacin	15 (62.5%)	14 (58.3%)	0.875	0.74	0.0001
Gentamycin	7 (29.2%)	7 (29.2%)	0.9167	0.79	0.00
Trimethoprim Sulpha	17 (70.8%)	4 (16.7%)	0.4583	0.15	0.0799

^a^ phenotype-based resistance

^b^ whole genome sequence-based resistance

### Phylogeny and genome comparison

**Fig. 1:**
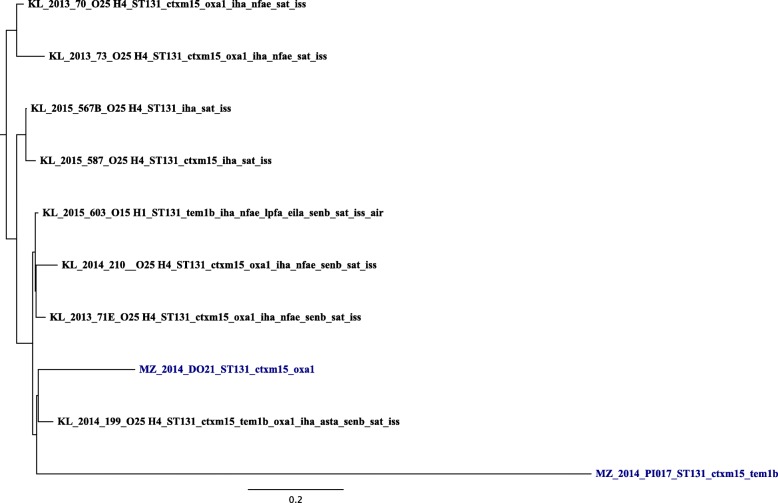
Phylogenetic analysis of 10 ST131 *E.coli* isolates showing serotypes, STs, resistance and virulence genes. KL stands for isolates sampled in the present study from patients hospitalised at a tertiary hospital in Kilimanjaro. MZ (blue colour) stands for isolates from companion and domesticated animals in Mwanza. The MZ sequences were downloaded as raw reads from European Nucleotide Archive (ENA) under the project number PRJEB12335. Number next to KL and MZ is date (year) of sampling or *E. coli* isolation. CTXM15 stands for *bla*_*CTX-M-15*_, OXA1 for *bla*_*OXA-1*_ and TEM1 for *bla*_*TEM-1*_

**Fig. 2:**
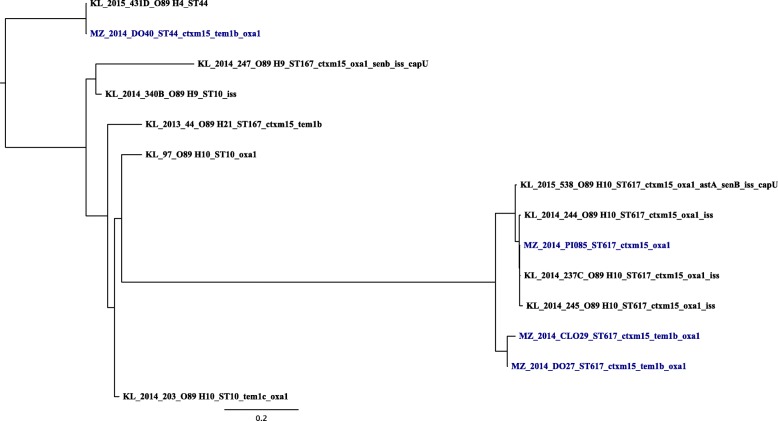
Phylogenetic analysis of 14 ST10 clonal complex (ST10, ST44, ST167, ST617) *E.coli* isolates showing serotypes, STs, resistance and virulence genes. KL stands for stands for isolates from patients hospitalised at a tertiary hospital in Kilimanjaro. MZ (blue colour) stands for isolates from companion and domesticated animals in Mwanza. The MZ sequences were downloaded as raw reads from European Nucleotide Archive (ENA) under the project number PRJEB12335. Number next to KL and MZ is date (year) of sampling or *E. coli* isolation. CTXM15 stands for *bla*_*CTX-M-15*_, OXA1 for *bla*_*OXA-1*_ and TEM1 for *bla*_*TEM-1*_

The observed minimum and maximum SNPs difference between one isolate and another in a pairwise genome comparison for ST131 were 234 and 10,425 respectively (Additional file 1: Table S1). The tree topology for ST131 showed one isolate segregating very distinctly from the rest (Fig. 1). For ST10 clonal complex the observed minimum and maximum SNPs difference between one isolate and another were 157 and 35,103 respectively (Additional file 2: Table S2). Looking at the tree for ST10 clonal complex (Fig. 2), limited pattern of the *E. coli* isolates was observed. In this tree, with the exception of ST617 in the middle clade of the ST10 clonal complex tree, all other STs making this complex occurred across the trees with little apparent segregation. Furthermore, the resistance and virulence genes were spread almost universally across both phylogenetic trees.

## Discussion

The present study revealed a high diversity of *E. coli* strains circulating in KCMC hospital settings as measured by Multi-Locus Sequence Typing and Serotyping. However, strains belonging to O25:H4-ST131 and O89:H10-ST617 (ST10 clonal complex) were found to predominate. These findings are similar to the findings from other continents describing the spread and predominance of these endemic clones in health facilities [31–34]. The observed clonal diversity in this hospital may suggest sporadic introductions of diverse strains into the hospital from the community. To explore whether or not similar STs were clonally related, SNP difference between isolates and phylogenetic analysis suggested the existence of multiple clones of *E. coli* in these settings. A similar clonal diversity was observed when STs from the present study were compared to similar STs of *E. coli* isolates from companion and other domesticated animals in Mwanza, north western Tanzania.

Overall, levels of antimicrobial resistance in *E. coli* isolates were observed to be high. Trimethoprim/sulphamethoxazole (co-trimoxazole) resistance was observed to be lower (70%) than the one (93%) found by Seni et al. [30], but in the present study on average 54–60% resistance to amoxicillin-clavulanate, ampicillin, ceftazidime, ceftriaxone, and ciprofloxacin was higher than that reported by Seni et al. [30]. Strong agreement (81–100%) between sequence and phenotype-based resistance to all drugs tested was observed. However, trimethoprim/sulphamethoxazole revealed moderate agreement (45.8%) between the two methods. The phenotypically determined resistance to trimethoprim/sulphamethoxazole was higher than sequence-based resistance. The plausible explanations for the observed difference could be that our analysis used only known resistance genes and did not include point mutations. Also, resistance in gram negative bacteria including *E. coli* is multifactorial and not all genes involved in resistance mechanisms have been uncovered.

Following analysis of resistance genes, over 70% of the *E. coli* strains carried genes encoding beta-lactamases, with *bla*_*OXA-1*_ being predominant (50%) followed by bla_CTX*-M-15*_ (44.7%) and *bla*_*TEM*-1B_ (31.6%). The prevalence of bla_CTX*-M-15*_ found in the present study contrasts with Manyahi et al. [18] who found bla_CTX*-M-15*_ as the most prevalent gene (90.6%) in a tertiary hospital in Dar es Salaam. Nonetheless, similar to the present study, O25:H4-ST131 was reported to be a major cause of MDR *E. coli* infections [35]. The proportion (70%) of *E. coli* strains carrying genes encoding beta-lactamases, correlates in this study with a high (50%) presence of *aac(6’)Ib-cr* encoding ciprofloxacin resistance enzymes. Although *aac(6’)Ib-cr* encodes low level ciprofloxacin resistance by itself (as well as aminoglycoside resistance), and usually requires additional mutations (e.g. in chromosomal *gyrA* or *parC*) to confer high level resistance, it is a threat to ciprofloxacin which in our settings is one of the most prescribed drugs. Other studies in the US [36, 37], Brazil [38] and Korea [34] have documented similar findings to the present study of the co-carriage of *aac(6’)Ib-cr*, ESBL genes and the existence of ciprofloxacin resistance in *E. coli* ST131. Further, an agreement between the presence or absence of *aac(6’)Ib-cr* and phenotypic resistance results in particular ciprofloxacin was noted by Madoshi et al. [39] who characterised *E. coli* isolates from healthy cattle and cattle attendants in Morogoro, Tanzania [39]. Among other fluoroquinolones, ciprofloxacin is one of the most prescribed antibiotics in Tanzania. Plausibly this explains the observed linkage between ciprofloxacin resistance and carriage of *aac(6’)Ib-cr* and CTX-M ESBL [31]. The linkage between quinolone resistance and sub-lineages of ST131 has been recently explored by Zakour et al. [40] whereby this work suggested that quinolone use is associated with the acquisition of virulence and fluoroquinolone resistance determinants and expansion of clade C2/H30-Rx of quinolone-resistant ST131. Also a gene (*mph(A)*) conferring resistance to macrolides including erythromycin and azithromycin was detected in a high proportion. The presence of the *mph(A)* in *E. coli* isolates of the current study was 50%, and thus, higher than the 13% that was found in *E. coli* from 5 countries from 4 continents by Nguyen et al. [41]. High exposures to erythromycin and azithromycin could be one possible reason leading to emergence of resistance to macrolides [42].

Furthermore, the present study noted a high proportion (73.7%) of *dfrA* genes encoding trimethoprim resistance enzymes similar to findings by Madoshi et al. [39] in Morogoro, Tanzania. Nonetheless, the present study proportion of *dfrA* genes was relatively higher than that (43%) found in *E. coli* isolates from healthy college students in Ghana and Nigeria in 2005 and 2009 [43]. The observed proportion difference could be explained by the fact that resistance in hospital-based studies is relatively higher than in community-based ones. Another reason could be that the present study was hospital-based whereas the West African study population were healthy individuals.

The present study also characterised VFs in all *E. coli* isolates. The VFs that facilitate *E. coli* and host cells or *E. coli* and *E. coli* adherence including long polar fimbriae (*lpfA*), adhesin (*iha*) and intimin (*eae*) were predominant among the circulating *E. coli* O25:H4-ST131 strains. These VFs have been identified as suggestive of virulent serotypes and may be used as reliable markers for the identification of pathogenic *E. coli* [44]. Another prevalent group of VFs this study noted were those responsible for *E. coli* immune evasion by increasing serum survival (*iss*). Although the predominance of *iss* in the present study was from *E. coli* isolated from hospitalised patients, in Mwanza north-western Tanzania, Msahana et al. [45] noted a similar pattern of *iss* in *E. coli* that were community-acquired. In this study we observed no clear correlation or pattern of VFs with the clinical findings or patient’s outcomes. However, colonisation events rather than infections were common as wound or pus swab and stool specimens constituted the majority of strains studied. The observed existence of multiple VFs further underlines the exceptional ability of *E. coli* in colonising or causing infections to a wide range of hosts and niches.

Potential clinical implications for the obtained results are that caution should be taken when interpreting and utilising microbiology results especially when *E. coli* is isolated in LMICs as most often *E. coli* is regarded of low threat. Contrary to that notion, these findings highlight that *E. coli* should not be regarded non-pathogenic until pathogenic and antimicrobial resistance determinants have been truly confirmed absent. Additionally, the WGS-based findings hint on the existence of nosocomial transmissions in the hospital thus prompting the formulation of pragmatic antimicrobial stewardships and infection prevention and control initiatives.

We understand and acknowledge the limitations of the present study. First, the analysis was performed on a small number of *E. coli* isolates, which may limit generalisation of the findings. With such a small number of the isolates analysed, it is important to point out that another limitation that this work was likely to suffer from was the lack of deeper statistical analysis to correlate the isolates resistance and virulence findings with patients characteristics (age, gender, ward, room, specimen) Third, the existence of genes encoding different resistance and virulence factors is only indicative of the genes present in the isolates. On the other hand, RNA sequencing which is an avenue for future studies could have given a strong evidence of expression levels of genes encoding different resistance and virulence factors.

## Conclusion

The observed high levels of *E. coli* diversity in terms of their antimicrobial resistance genes, serotypes and virulence genes underlines the necessity for concerted efforts to routinely screen all bacterial isolates of clinical importance especially in tertiary health care facilities. WGS use for laboratory-based surveillance can be an effective early warning system for emerging pathogens and resistance mechanisms in LMICs. The information generated in this study will not only provide updates on levels of virulence and antimicrobial resistance at hospital level but will also be used as a basis in formulating pragmatic antimicrobial stewardships and infection prevention and control initiatives.

## Additional files

Additional file 1: **Table S1.** A pairwise genome comparison matrix of SNP differences for *E. coli* ST131. (XLS 34 kb)

Additional file 2: **Table S2.** A pairwise genome comparison matrix of SNP differences for *E. coli* ST10 clonal complex. (XLS 29 kb)
